# Green design of biodegradable packaging films *via* renewable coffee waste and PVA matrix tuning

**DOI:** 10.1039/d5ra08990e

**Published:** 2026-03-26

**Authors:** Edy Supriyanto, Muhammad Rizky Ramadhani, Dwi Sabda Budi Prasetya, Stefano Akbar, Sri Subekti, Aryo Fajar Sunartomo, K. Triyana

**Affiliations:** a Department of Physics, Faculty of Mathematics and Natural Sciences, University of Jember Kalimantan Street 37 Jember 68121 East Java Indonesia edysupriyanto@unej.ac.id; b Biomedical Engineering, Faculty of Science and Technology, PGRI University Yogyakarta Yogyakarta 55182 Indonesia; c Agricultural Extension Study Program, Faculty of Agriculture, University of Jember Kalimantan Street 37 Jember 68121 East Java Indonesia; d Department of Physics, Faculty of Mathematics and Natural Sciences, Gadjah Mada University North Sekip Yogyakarta 55281 Indonesia

## Abstract

Biodegradable packaging materials derived from renewable resources have attracted increasing attention as sustainable alternatives to conventional petroleum-based plastics. In this study, biodegradable composite films based on poly(vinyl alcohol) (PVA) reinforced with spent coffee grounds (SCG), an abundant agro-industrial waste, were successfully fabricated *via* a simple and environmentally benign water-based solution casting method. The effect of PVA matrix concentration (6–14% w/v) was systematically investigated to elucidate its role as a key structural parameter governing film formation, intermolecular interactions, thermal behaviour, and mechanical performance. Fourier transform infrared (FTIR) analysis provided direct evidence of hydroxyl-driven intermolecular interactions between PVA chains and lignocellulosic components of SCG, confirming the formation of hydrogen-bonded networks within the composite films. Thermogravimetric analysis (TGA) revealed a predictable multi-step degradation mechanism, with the main decomposition occurring between 250 and 500 °C and the formation of residual carbonaceous char at higher temperatures due to lignin-rich SCG fractions. Differential scanning calorimetry (DSC) showed a distinct melting transition around 170 °C and a degradation-related thermal event above 300 °C, indicating that crystalline domains and melting stability of PVA were preserved after SCG incorporation. Mechanical testing demonstrated that PVA concentration strongly influenced tensile behaviour. The composite films exhibited ductile deformation with high elongation at break (300–890%). An optimum formulation was identified at 12% PVA, which achieved the best balance between strength and flexibility, reaching a maximum tensile strength of 1.70 MPa and elongation at break of 889%. At higher PVA concentration (14%), excessive solution viscosity reduced filler dispersion homogeneity, leading to a slight decrease in tensile strength while maintaining high flexibility. Overall, this work highlights the importance of polymer matrix concentration as a critical design parameter beyond filler loading alone and demonstrates the potential of spent coffee grounds as a renewable, circular-economy reinforcement for sustainable PVA-based biodegradable packaging films.

## Abstract 2

Biodegradable films reinforced with coffee waste and variably tuned PVA matrix.

## Graphical abstract

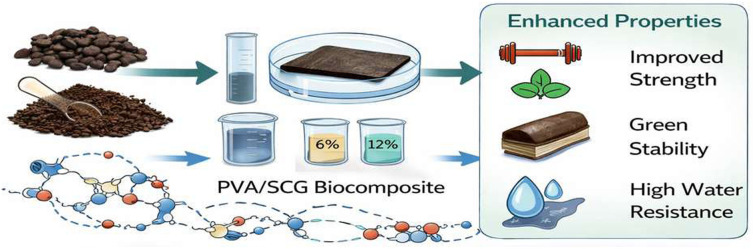

## 1. Introduction

The extensive use of conventional petroleum-based plastics in packaging applications has resulted in serious environmental challenges, including long-term persistence in ecosystems, microplastic pollution, and increasing landfill accumulation. These materials exhibit excellent durability and low cost; however, their resistance to natural degradation has raised significant concerns regarding environmental sustainability and waste management.^1–3^ Consequently, the development of biodegradable and environmentally friendly packaging materials derived from renewable resources has become a critical research priority.

Among synthetic biodegradable polymers, poly(vinyl alcohol) (PVA) has attracted considerable attention due to its excellent film-forming ability, transparency, flexibility, biocompatibility, and superior oxygen-barrier properties.^4–6^ PVA is produced by the hydrolysis of poly(vinyl acetate), resulting in polymer chains rich in hydroxyl groups capable of forming strong intermolecular hydrogen bonds. While these interactions enhance cohesive strength, they also impart pronounced hydrophilicity, leading to high moisture sensitivity and partial solubility in aqueous environments.^7–9^ This inherent limitation restricts the application of neat PVA films in humid or water-contact packaging systems.^10^

To overcome these drawbacks, various modification strategies have been proposed, including chemical cross-linking, polymer blending, and incorporation of reinforcing fillers. Chemical cross-linking using agents such as glutaraldehyde or citric acid effectively improves water resistance and mechanical stability; however, the use of additional chemicals may compromise biodegradability and raise toxicity concerns.^11,12^ Blending PVA with natural polymers such as starch, chitosan, or cellulose has also been widely explored, yielding partially improved performance but often at the expense of mechanical strength or moisture stability.^13–15^

Recently, increasing attention has been directed toward the use of renewable waste-derived fillers as a sustainable reinforcement strategy. Among these, spent coffee grounds (SCG) represent a particularly attractive candidate. Globally, millions of tons of SCG are generated annually as waste from coffee consumption, with the majority disposed of in landfills, contributing to greenhouse gas emissions.^16–18^ Chemically, SCG is rich in lignocellulosic components, including cellulose, hemicellulose, lignin, and phenolic compounds, which provide abundant hydroxyl and aromatic functional groups capable of interacting strongly with polymer matrices.^19–21^

Several studies have demonstrated that spent coffee grounds (SCG) can function as an effective reinforcement in biodegradable polymer systems. Asrofi *et al.*^5^ reported that the incorporation of SCG into PVA–starch biocomposite films significantly improved tensile strength, which was attributed to enhanced interfacial adhesion between the lignocellulosic filler and the polymer matrix. Similarly, Mendes and co-workers^22^ observed improved thermal stability and barrier performance in SCG-modified pectin films, although the mechanical properties were strongly influenced by filler dispersion and moisture sensitivity. These findings highlight the potential of SCG as a multifunctional filler while emphasising the importance of matrix structure and processing conditions in determining the overall performance of biodegradable polymer composites.

Despite these advances, most previous investigations have primarily focused on varying filler content while maintaining a constant polymer matrix concentration. However, polymer concentration plays a fundamental role in governing solution viscosity, chain entanglement density, film compactness, and free-volume distribution.^23–25^ At low polymer concentrations, insufficient chain overlap leads to porous and mechanically fragile films. Conversely, increasing polymer concentration promotes stronger intermolecular interactions and improves structural integrity. Beyond an optimal concentration, however, excessive viscosity can hinder filler dispersion and promote microstructural heterogeneities that act as stress concentrators within the composite matrix.^26,27^

In PVA-based systems, the balance between mechanical performance and water interaction is particularly critical. Strong hydrogen bonding and increased crystallinity can enhance tensile strength and reduce solubility, but excessive network rigidity may compromise flexibility. Conversely, high hydrophilicity promotes ductility and swelling while reducing dimensional stability and moisture resistance.^28–31^ A systematic understanding of how PVA matrix concentration influences these competing effects in SCG-reinforced films remains limited.

Beyond mechanical reinforcement, SCG also offers functional advantages relevant to active packaging applications. Phenolic compounds naturally present in SCG have been reported to exhibit antioxidant and antimicrobial activity, while the carbon-rich structure can contribute to ultraviolet (UV) light attenuation and improved barrier behaviour.^31–33^ Furthermore, the utilisation of SCG as a filler aligns with circular-economy strategies by converting abundant agro-industrial waste into value-added functional materials for sustainable polymer systems.^34,35^

**Fig. 1:**
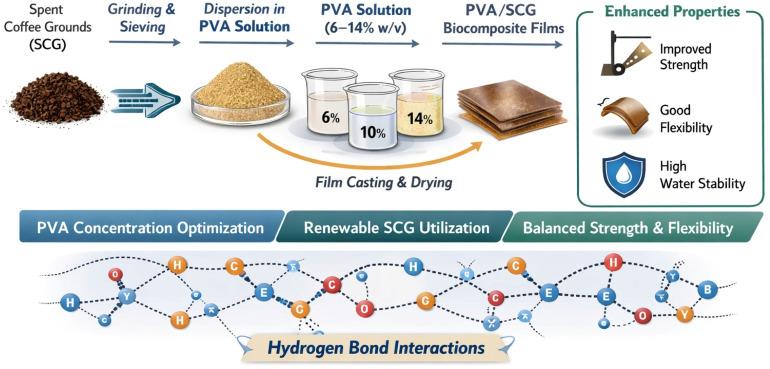
Schematic illustration of the research concept: conversion of spent coffee grounds (SCG) into fine lignocellulosic powder, dispersion in poly(vinyl alcohol) (PVA) solution at various polymer concentrations (6–14% w/v), film casting, and resulting enhancement of mechanical performance and water stability through hydrogen-bond-driven intermolecular interactions.

Therefore the objective of this study is to systematically investigate the effect of PVA matrix concentration (6–14% w/v) on the structural, thermal, and mechanical properties of PVA/SCG composite films prepared using a simple water-based solution. Particular emphasis is placed on elucidating the role of polymer concentration as a critical design parameter governing intermolecular interactions, thermal stability, and structure–property relationships in SCG-reinforced biodegradable films. A schematic overview of the research concept is presented in Fig. 1.

This work provides a systematic understanding of how PVA matrix concentration influences the structural and functional performance of SCG-reinforced biodegradable films. By integrating renewable waste-derived fillers with controlled polymer network formation, the study contributes to the development of sustainable polymer–biowaste composites for environmentally friendly packaging applications.

## 2. Experimental

### 2.1 Materials

Poly(vinyl alcohol) (PVA) with an average molecular weight of 89 000–98 000 g mol^−1^ and a degree of hydrolysis of 87–89% was purchased from Sigma-Aldrich and used without further purification. Spent coffee grounds (SCG) were collected from a local coffee shop in Jember, Indonesia, immediately after the brewing process to minimise microbial degradation. Distilled water was used as the sole solvent throughout the study to comply with green-chemistry principles. Similar grades of PVA and lignocellulosic coffee waste have been widely employed in biodegradable film and biocomposite studies.^1–3^

The collected SCG was washed repeatedly with distilled water to remove residual soluble compounds and coffee oils, followed by oven drying at 105 °C for 24 h. The dried SCG was ground and sieved to obtain particles smaller than 80 mesh (≈180 µm). This pre-treatment step was adopted to improve filler dispersion and interfacial contact with the polymer matrix, as reported in previous SCG-based composite studies.^4,5^

### 2.2 Preparation of SCG-reinforced PVA films

**Fig. 2:**
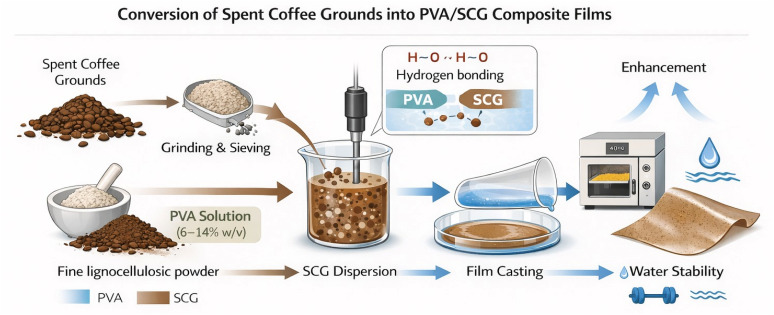
Schematic illustration of the preparation of PVA/SCG composite films *via* water-based solution casting, including SCG pre-treatment, PVA dissolution, filler dispersion, and film formation.

PVA/SCG composite films were fabricated *via* a water-based solution casting method with systematic variation of PVA concentration (6, 8, 10, 12, and 14% w/v) to investigate the influence of polymer matrix density on film properties (Fig. 2).

Initially, the required amount of PVA powder was gradually added to distilled water and heated to 90 °C under continuous magnetic stirring for 1 h until a clear and homogeneous viscous solution was obtained. This temperature was selected to ensure complete dissolution of PVA without thermal degradation, consistent with established protocols for PVA film preparation.^6,7^

Pre-treated SCG powder was then incorporated into the warm PVA solution at a fixed loading of 1 wt% relative to the mass of PVA. The mixture was stirred at 600 rpm for 30 min, followed by ultrasonication at 40 kHz for 15 min to enhance filler dispersion and minimise particle agglomeration. Similar dispersion strategies have been shown to significantly improve the homogeneity of lignocellulosic-filled polymer films.^8,9^

The resulting suspension was degassed to remove entrapped air bubbles and cast onto clean glass Petri dishes (10 cm diameter) to obtain films with an average thickness of approximately 0.15 mm. Drying was carried out in a convection oven at 40 °C for 24 h to allow slow solvent evaporation and reduce internal stress formation. The dried films were subsequently conditioned at 25 ± 2 °C and 50 ± 5% relative humidity for at least 48 h prior to characterisation.

The films were coded according to PVA concentration as PVA 6, PVA 8, PVA 10, PVA 12, and PVA 14. Films prepared with PVA concentrations up to 12% were transparent and flexible, while those prepared at 14% PVA appeared slightly opaque, attributed to increased solution viscosity and reduced SCG dispersion.

### 2.3 Characterisation methods

#### 2.3.1 Fourier transform infrared (FTIR) spectroscopy

FTIR spectroscopy was employed to analyse chemical structure and intermolecular interactions within the composite films. Spectra were recorded in the range of 4000–500 cm^−1^ at a resolution of 4 cm^−1^ with 32 scans per sample. Particular attention was given to changes in hydroxyl stretching and polysaccharide-related bands to evaluate hydrogen bonding between PVA and SCG components.^10,11^

#### 2.3.2 Thermogravimetric analysis (TGA)

Thermal stability and degradation behaviour were investigated using thermogravimetric analysis. Approximately 5–10 mg of each sample was heated from room temperature to 700 °C at a heating rate of 10 °C min^−1^ under a nitrogen atmosphere. The mass-loss profiles were analysed to identify degradation stages associated with PVA and lignocellulosic SCG fractions.^12,13^

#### 2.3.3 Differential scanning calorimetry (DSC)

Differential scanning calorimetry was used to determine thermal transitions of the composite films. Samples weighing approximately 5 mg were sealed in aluminium pans and heated from 30 to 400 °C at a rate of 10 °C min^−1^ under nitrogen. Melting temperature, transition onset, and enthalpy changes were obtained from the thermograms.^14^

#### 2.3.4 Density measurement

Film density was determined according to ASTM D792 using the mass-to-volume ratio. Film mass was measured using an analytical balance, while volume was calculated from measured thickness and surface area. Reported values represent the average of at least five specimens per formulation.^15^

#### 2.3.5 Moisture content

Moisture content was measured gravimetrically by drying pre-conditioned film samples at 105 °C for 24 h. Moisture content was calculated as the percentage mass loss relative to the initial mass. Measurements were performed in triplicate, following methods commonly used for biodegradable polymer films.^16^

#### 2.3.6 Water solubility

Water solubility was evaluated by immersing dried film samples (≈0.1 g) in 50 mL of distilled water at 25 °C for 24 h under gentle agitation. The remaining insoluble fraction was recovered, dried, and weighed. Solubility was calculated as the percentage of dissolved material, following previously reported protocols for PVA-based films.^17^

#### 2.3.7 Water absorption

Water absorption capacity was assessed by immersing dried film specimens in distilled water at room temperature for 24 h. Samples were gently blotted and weighed, and water absorption was calculated as the percentage increase in mass relative to the dry state. Visual inspection confirmed that no film disintegration occurred during immersion.^18^

#### 2.3.8 Mechanical testing

Mechanical properties were measured using a universal testing machine in accordance with ASTM D882. Film specimens (80 mm × 10 mm) were tested at a gauge length of 50 mm and a cross-head speed of 5 mm min^−1^ under ambient conditions (25 ± 2 °C, 50 ± 5% RH). Tensile strength, elongation at break, and Young's modulus were calculated from the stress–strain curves. Average values were obtained from at least five specimens per formulation.^19,20^

### 2.4 Statistical analysis

All experiments were conducted at least in triplicate, and results are presented as mean ± standard deviation. Statistical significance among different formulations was evaluated using one-way analysis of variance (ANOVA) followed by Tukey's *post hoc* test, with *p* < 0.05 considered statistically significant.

### 2.5 Safety and environmental considerations

All experimental procedures were performed using non-toxic, water-based solvents and renewable raw materials in accordance with green-chemistry and sustainability principles. Residual SCG waste was dried and disposed of as biodegradable organic material. No hazardous chemicals were used during film preparation or characterisation, minimising environmental impact and ensuring laboratory safety.

## 3. Results and discussion

### 3.1 Film appearance and morphology

**Fig. 3:**
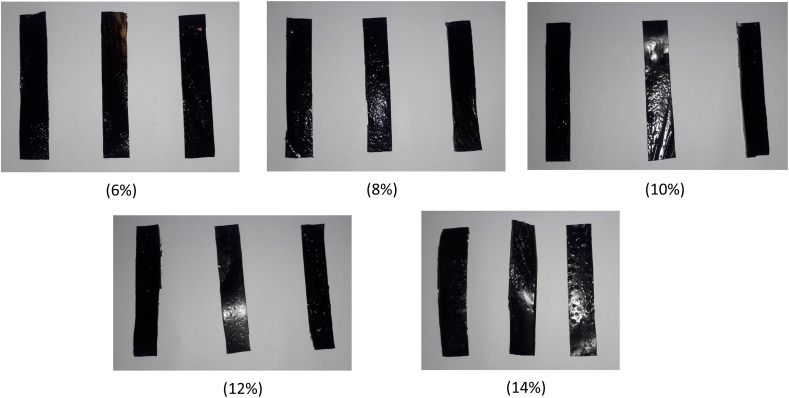
Photographs of PVA/SCG composite films prepared with different PVA concentrations (6–14% w/v), illustrating changes in transparency and visual homogeneity.

All PVA/SCG composite films produced in this study were self-supporting, flexible, and could be peeled easily from the casting substrate without visible cracking or tearing. Films prepared with PVA concentrations between 6% and 12% exhibited good transparency and visual homogeneity, whereas films prepared at 14% PVA appeared slightly opaque. This opacity is attributed to the increased viscosity of the casting solution at high polymer concentrations, which can hinder uniform dispersion of SCG particles and promote microstructural heterogeneities that scatter incident light. Similar behaviour has been reported in highly concentrated PVA and lignocellulosic-filled polymer systems (Fig. 3).^1,2^

The incorporation of SCG imparted a light brown coloration to all films, originating from phenolic compounds naturally present in coffee residues. Such coloration may provide partial ultraviolet (UV) shielding, which is advantageous for packaging applications requiring light protection.^3,4^ Optical observations suggest that films prepared at intermediate PVA concentrations exhibit improved surface compactness compared to those prepared at low or excessively high concentrations, indicating a balance between polymer chain entanglement and filler dispersion.

### 3.2 FTIR analysis of intermolecular interactions

Fourier transform infrared (FTIR) spectroscopy was employed to provide direct molecular-level evidence of intermolecular interactions between the poly(vinyl alcohol) (PVA) matrix and the lignocellulosic components of spent coffee grounds (SCG).

**Fig. 4:**
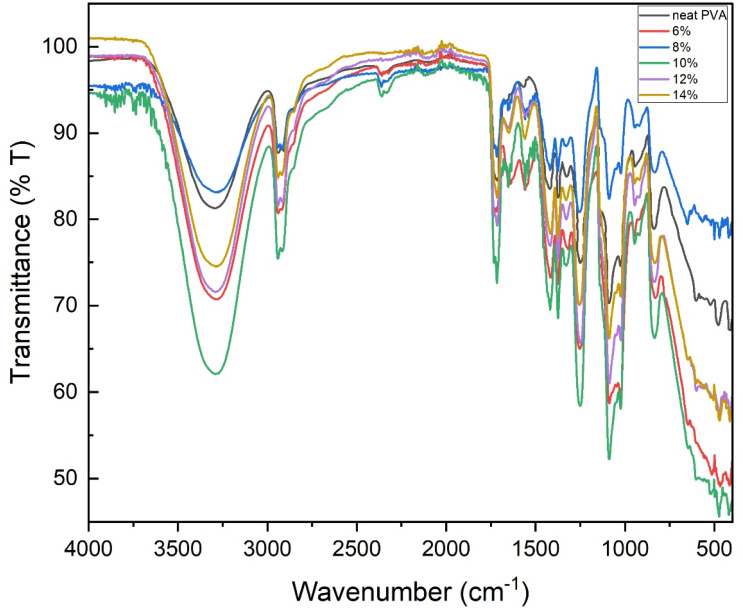
FTIR spectra of PVA/SCG composite films prepared with different PVA concentrations (6–14% w/v), highlighting hydroxyl stretching, carbonyl groups, and polysaccharide-related vibrations indicative of intermolecular hydrogen bonding.

Fig. 4 presents the FTIR spectra of composite films prepared with different PVA concentrations. All spectra exhibit the characteristic absorption features of hydroxyl-rich PVA-based systems. A broad absorption band in the range of 3200–3500 cm^−1^ is observed for all samples and is attributed to O–H stretching vibrations originating from both PVA chains and hydroxyl groups present in the cellulose and hemicellulose fractions of SCG. The pronounced broadening of this band indicates extensive hydrogen bonding within the composite network, confirming that hydroxyl-driven interactions govern matrix–filler interfacial adhesion.^5–7^

Absorption bands located around 2900–3000 cm^−1^ correspond to C–H stretching vibrations of the PVA backbone, indicating that the incorporation of SCG does not alter the fundamental hydrocarbon structure of the polymer. A noticeable band near 1700 cm^−1^ is assigned to C=O stretching vibrations associated with residual lignin and other carbonyl-containing compounds present in SCG, which may contribute to secondary intermolecular interactions with the PVA matrix.^8,9^

The fingerprint region between 1000 and 1200 cm^−1^ exhibits strong absorption bands related to C–O–C and C–O stretching vibrations characteristic of polysaccharide structures in SCG as well as PVA skeletal vibrations. With increasing PVA concentration, progressive changes in the intensity and shape of the O–H band are observed, indicating enhanced polymer chain entanglement and matrix cohesion. At the highest PVA concentration (14%), excessive solution viscosity during casting may hinder uniform SCG dispersion, consistent with the slight deterioration in mechanical performance discussed later.

### 3.3 Thermogravimetric analysis (TGA)

Thermogravimetric analysis (TGA) was carried out to evaluate the thermal stability and degradation behaviour of the PVA/SCG composite films, which is a critical requirement for biodegradable packaging materials subjected to processing or storage at elevated temperatures.

**Fig. 5:**
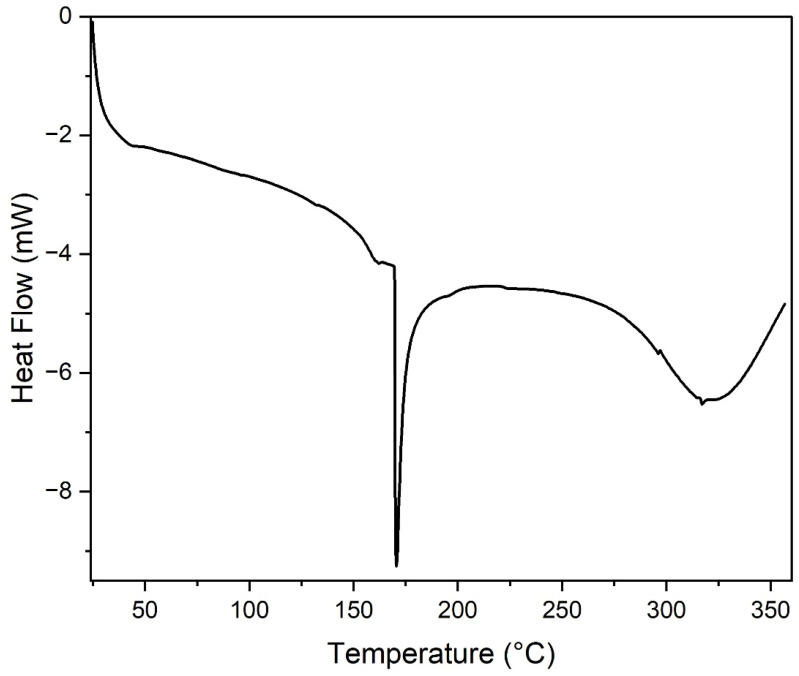
Thermogravimetric (TGA) curves of PVA/SCG composite films, showing multi-step degradation behaviour and residual char formation.

The TGA mass-loss profiles (Fig. 5) reveal a multi-step degradation mechanism reflecting the combined thermal responses of the PVA matrix and the lignocellulosic SCG filler. An initial minor mass loss below approximately 250 °C is attributed to the evaporation of physically adsorbed moisture and the release of low-molecular-weight volatile components, consistent with the hydrophilic nature of both PVA and SCG.^10,11^

The primary degradation stage occurs between 250 and 500 °C, where a sharp decrease in mass is observed. This region corresponds to thermal scission of the PVA backbone as well as degradation of hemicellulose and cellulose fractions in SCG. The retention of structural integrity up to this temperature range confirms that the composite films remain thermally stable under typical packaging and processing conditions.^12^

At temperatures above 500 °C, the degradation rate decreases significantly, with gradual mass loss observed up to 700 °C. This stage is associated with decomposition of thermally stable lignin fractions and the formation of residual carbonaceous char. The presence of char residue reflects the lignin-rich nature of SCG and has been widely reported in biomass-filled polymer composites.^13,14^

### 3.4 Differential scanning calorimetry (DSC)

Differential scanning calorimetry (DSC) was employed to investigate thermal transitions of the PVA/SCG composite films, including melting behaviour and degradation-associated events.

**Fig. 6:**
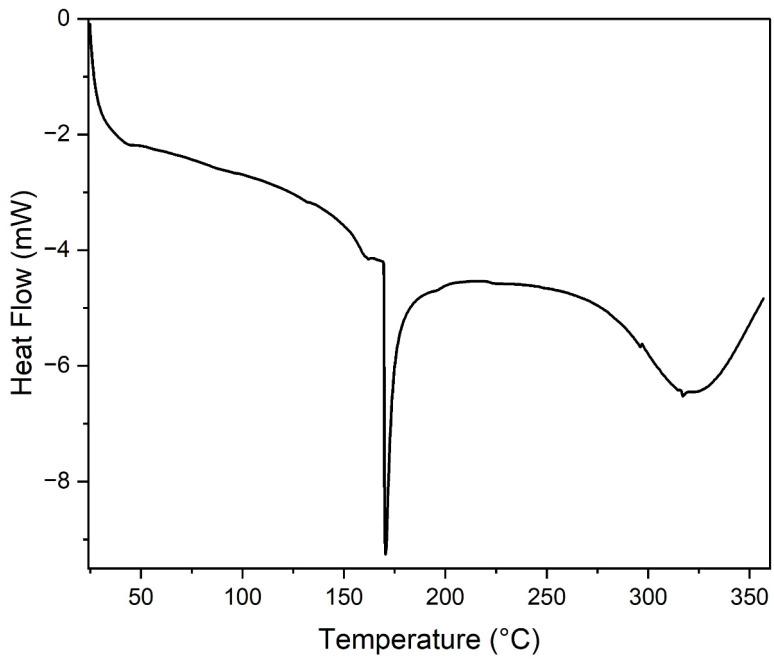
DSC thermograms of PVA/SCG composite films showing melting behaviour and high-temperature degradation-related thermal events.

The DSC thermograms (Fig. 6) reveal three distinct thermal events. A minor endothermic transition occurring between approximately 154 and 168 °C is attributed to structural relaxation or pre-melting rearrangement within amorphous regions of the PVA matrix. The low enthalpy associated with this transition indicates limited molecular reorganisation.^15^

The primary melting transition is observed between 169 and 175 °C, with a peak around 170.5 °C. The substantial enthalpy change confirms the presence of crystalline domains within the composite films, demonstrating that SCG incorporation does not suppress PVA crystallisation. Instead, SCG particles may act as heterogeneous nucleation sites, promoting local crystalline organisation through interfacial interactions.^16,17^

A third broad endothermic event observed above 300 °C corresponds to irreversible thermal degradation of the polymer backbone and lignocellulosic SCG components, in agreement with the main decomposition stage identified by TGA.

### 3.5 Mechanical properties

The mechanical performance of biodegradable packaging films is a key factor governing their practical applicability. Tensile testing was therefore conducted to evaluate the influence of PVA concentration on strength, ductility, and stiffness of the PVA/SCG composite films.

**Fig. 7:**
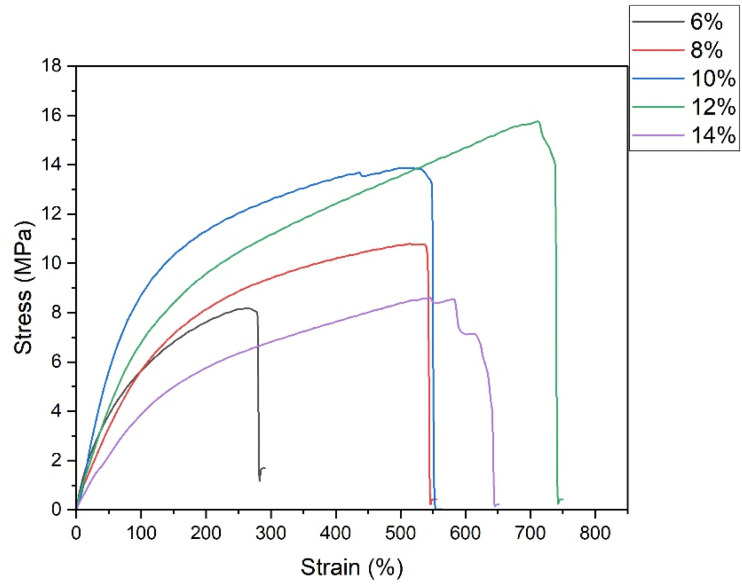
Representative stress–strain curves of PVA/SCG composite films prepared with different PVA concentrations (6–14% w/v).

Representative stress–strain curves (Fig. 7) indicate that all composite films exhibit ductile deformation behaviour, confirming that the incorporation of SCG does not induce brittle failure within the investigated concentration range. The ultimate tensile strength increases significantly with increasing PVA concentration from 6% to 12%, reflecting enhanced polymer chain entanglement and improved stress transfer across the matrix–filler interface. Similar trends have been reported for PVA composites reinforced with lignocellulosic fillers.^18–20^

**Fig. 8:**
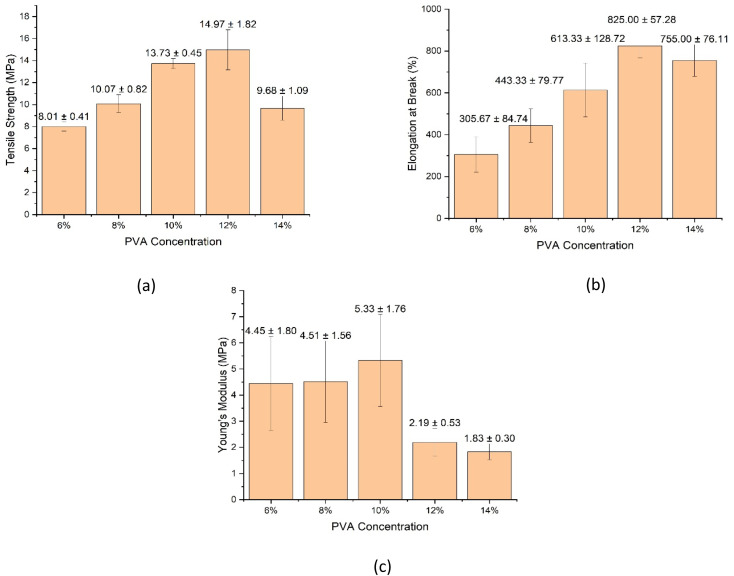
Tensile properties of PVA/SCG composite films as a function of PVA concentration: (a) tensile strength, (b) elongation at break, and (c) Young's modulus.

The highest tensile strength and yield strength are obtained for the 12% PVA formulation, indicating an optimal balance between matrix cohesion and filler dispersion. At 14% PVA concentration, a slight reduction in tensile strength is observed, attributed to reduced dispersion homogeneity caused by excessive solution viscosity during casting, which can generate local stress concentrators (Fig. 8).^21^

All composite films demonstrate remarkably high elongation at break, ranging from approximately 300% to nearly 900%. The highest elongation is observed for the 12% formulation, indicating a synergistic balance between strength and flexibility. Films prepared with 14% PVA maintain high elongation values, suggesting suitability for applications requiring highly extensible packaging materials.

Young's modulus decreases with increasing PVA concentration, indicating a transition from relatively rigid films toward more flexible polymer networks. This behaviour is consistent with increased chain mobility at higher polymer contents.^22^

### 3.6 Structure–property relationships and durability considerations

The combined results demonstrate that intermolecular hydrogen bonding between hydroxyl groups of PVA and lignocellulosic components of SCG governs the structure–property relationships of the composite films. Increasing PVA concentration enhances chain entanglement, matrix densification, and interfacial adhesion, resulting in improved mechanical strength and thermal stability up to an optimal concentration range.

Beyond this optimum, excessive solution viscosity limits filler dispersion and introduces microstructural heterogeneities that act as stress concentrators, leading to a slight reduction in tensile strength. From a durability perspective, while SCG is an organic residue that may promote microbial growth under humid conditions, it also contains phenolic compounds reported to exhibit antimicrobial and antioxidant activity. Proper drying, controlled storage, and potential surface treatments are therefore expected to mitigate long-term degradation issues.^23,24,25^

Overall, an optimal PVA concentration window around 10–12% achieves a synergistic balance between mechanical robustness, flexibility, and thermal stability, highlighting the potential of spent coffee grounds as a renewable reinforcement for sustainable biodegradable packaging films.

## 4. Conclusion

Biodegradable composite films based on poly(vinyl alcohol) (PVA) reinforced with spent coffee grounds (SCG) were successfully fabricated *via* a simple, environmentally friendly water-based solution casting method. The systematic variation of PVA matrix concentration (6–14% w/v) enabled a comprehensive evaluation of its role as a critical design parameter governing film structure, intermolecular interactions, thermal stability, and mechanical performance.

Spectroscopic analysis using FTIR provided direct molecular-level evidence of hydroxyl-driven intermolecular interactions between PVA chains and lignocellulosic components of SCG, confirming the formation of hydrogen-bonded networks within the composite films. Thermal characterisation through TGA revealed a predictable multi-step degradation mechanism, with the main decomposition occurring between 250 and 500 °C and residual char formation at higher temperatures due to the lignin-rich nature of SCG. Complementary DSC analysis demonstrated that the incorporation of SCG does not suppress PVA crystallisation, as indicated by a distinct melting transition around 170 °C, while major thermal degradation occurred only above 300 °C.

Mechanical testing showed that PVA concentration strongly influences tensile behaviour. Increasing polymer content enhanced chain entanglement and stress transfer, leading to improved tensile strength and ductility up to an optimum at 12% PVA. This formulation exhibited the best balance between strength and flexibility, combining high tensile strength with elongation at break approaching 900%. At higher PVA concentration (14%), excessive solution viscosity reduced filler dispersion homogeneity, resulting in a slight decrease in tensile strength while maintaining high flexibility.

Overall, the results demonstrate that polymer matrix concentration is a key parameter in the design of PVA/SCG composite films, beyond filler loading alone. An optimal PVA concentration window around 10–12% achieves a synergistic balance between mechanical robustness, flexibility, and thermal stability, making these films promising candidates for sustainable biodegradable packaging applications.

This study highlights the potential of spent coffee grounds as a renewable circular-economy reinforcement for polymer-based films and provides practical insight into structure–property optimisation of biowaste-derived composites. Future work will focus on benchmarking against neat PVA films, long-term durability under humid and microbial exposure conditions, and the development of multifunctional packaging systems incorporating the intrinsic antioxidant and antimicrobial properties of SCG.

## Author contributions

Edy Supriyanto: conceptualisation, methodology, supervision, and writing – review & editing. Muhammad Rizky Ramadhani: investigation, data curation, formal analysis, and writing – original draft. Dwi Sabda Budi Prasetya: visualization, validation, and writing – review & editing. Stefano Akbar: experimental work, data analysis, and resources. K. Triyana: expert consultation, validation, and critical review of the manuscript. Sri Subekti: data analysis and experimental work. Aryo Fajar Sunartomo: data analysis and resources.

## Conflicts of interest

The authors declare no competing financial interest.

## Supplementary Material

RA-016-D5RA08990E-s001

## Data Availability

The data that support the findings of this study are available from the corresponding author upon reasonable request. Supplementary information (SI): additional supporting data related to the fabrication and characterization of the PVA/SCG composite films. Specifically, it contains figures, detailed experimental procedures, and additional data supporting the FTIR, thermal (TGA/DSC), and mechanical analyses presented in the main manuscript. These materials are provided to support the reproducibility and transparency of the reported results. is available. See DOI: https://doi.org/10.1039/d5ra08990e.
